# Designing and Evaluating Bamboo Harvesting Methods for Local Needs: Integrating Local Ecological Knowledge and Science

**DOI:** 10.1007/s00267-016-0702-6

**Published:** 2016-04-25

**Authors:** András Darabant, Prem Bahadur Rai, Christina Lynn Staudhammer, Tshewang Dorji

**Affiliations:** Department of Forest and Soil Sciences, University of Natural Resources and Life Sciences, Peter Jordan Strasse 82, 1190 Vienna, Austria; Ugyen Wangchuk Institute for Conservation and Environmental Research, Lamai Goempa, Bumthang, Bhutan; Department of Biological Sciences, University of Alabama, Box 870344, Tuscaloosa, AL 35487 USA

**Keywords:** *Dendrocalamus hamiltonii*, Bamboo silviculture, NTFP harvesting, Local ecological knowledge, Participatory action research

## Abstract

*Dendrocalamus hamiltonii,* a large, clump-forming bamboo, has great potential to contribute towards poverty alleviation efforts across its distributional range. Harvesting methods that maximize yield while they fulfill local objectives and ensure sustainability are a research priority. Documenting local ecological knowledge on the species and identifying local users’ goals for its production, we defined three harvesting treatments (selective cut, horseshoe cut, clear cut) and experimentally compared them with a no-intervention control treatment in an action research framework. We implemented harvesting over three seasons and monitored annually and two years post-treatment. Even though the total number of culms positively influenced the number of shoots regenerated, a much stronger relationship was detected between the number of culms harvested and the number of shoots regenerated, indicating compensatory growth mechanisms to guide shoot regeneration. Shoot recruitment declined over time in all treatments as well as the control; however, there was no difference among harvest treatments. Culm recruitment declined with an increase in harvesting intensity. When univariately assessing the number of harvested culms and shoots, there were no differences among treatments. However, multivariate analyses simultaneously considering both variables showed that harvested output of shoots and culms was higher with clear cut and horseshoe cut as compared to selective cut. Given the ease of implementation and issues of work safety, users preferred the horseshoe cut, but the lack of sustainability of shoot production calls for investigating longer cutting cycles.

## Introduction

Globally, more than 1.6 billion people depend on forests for their livelihoods to some degree (Angelsen and Wunder 2003), and this dependence is stronger in remote places with high levels of poverty (Sunderlin et al. 2008). In these areas, non-timber forest products (NTFPs) frequently play an important “safety-net” function for rural poor (Belcher and Schreckenberg 2007) and thus reduce risk. Moreover, the rate of return for NTFPs typically exceeds that generated by agricultural activities (Krishnankutty 2004; Pandit and Kumar 2010). Beyond subsistence, forest products also provide employment and income opportunities for local forest users (Arnold and Pérez 2001). Commercialization of NTFPs has been promoted for environmental conservation and livelihood support but often has failed to achieve these outcomes simultaneously (Arnold and Pérez 2001; Kusters et al. 2006). While marketing and sales are apparently the most important constraints (Marshall et al. 2003), the lack of sustainable utilization methods remains a key challenge to successful NTFP development (Belcher and Schreckenberg 2007; Wong et al. 2001). Initially successful NTFP-based cottage industries are frequently affected by declines in production resulting from over-harvesting or improper management of the resource base due to lack of appropriate management guidelines (Prommegger et al. 2005; Moktan et al. 2009).

Scientific knowledge on NTFPs is frequently limited as a result of the relatively recent public interest in these commodities and their great diversity. At the same time, community-based management relying on local ecological knowledge (LEK) has yielded positive outcomes in managing NTFPs (Rist et al. 2010). LEK, however, varies widely within user groups (Chalmers and Fabricius 2007) and may be limited to selected aspects of the ecology, growth conditions, and utilization of particular species and ecosystems (Ballard and Huntsinger 2006; Nath et al. 2015a). LEK on NTFPs that are traditionally harvested for subsistence is frequently limited to experiences with extensive, low intensity utilization. Improved marketing, sales, and transport opportunities open possibilities for commercialization of NTFPs (Marshall et al. 2003) and may lead to a change of the household strategy in managing NTFPs, from subsistence to supplementary or integrated management (Belcher et al. 2005). This shift of the household strategy inevitably results in higher intensity management, for which traditional resource management approaches may not be adequate, leading to over-harvesting and resource decline (Belcher and Schreckenberg 2007). In such situations, LEK may not provide adequate answers to complex, knowledge intensive practices, particularly if these have emerged recently (Ingram 2008). Co-production of knowledge between scientists and users may provide answers in such situations (Kainer et al. 2009; Kristjanson et al. 2009). Moreover, the scientific testing of LEK provides a possible way to integrate it into formal management plans (Ticktin and Johns 2002), which are often required by governments for recognizing traditional rights and community-based management (Leach et al. 1999). Harvesting regimes of NTFPs must maximize sustainable output of the target commodity, but they also need to fit the local socio-economic context. Participatory action research provides a useful framework to develop and test harvesting regimes integrating LEK and provides a platform for disseminating findings into practice (Ticktin et al. 2002).

Bamboos are versatile and weakly perishable non-timber forest products that are important sources of livelihood in many rural areas (Lobovikov et al. 2012). Throughout monsoonal Asia, bamboo culms are widely used for construction, fencing, and handicrafts, while bamboo shoots are an important dietary supplement of high nutritional value (Bhatt et al. 2005; Moktan et al. 2009). Additionally, the carbon sequestration potential of bamboo groves can further increase their livelihood contribution (Nath et al. 2015b). Bamboo-based local enterprises have successfully contributed to poverty alleviation (Zhu 2003; Moktan et al. 2009) and have large economic potential in the region (Bhatt et al. 2003). Accordingly, bamboo production within the legal framework of community forests also formed part of national development targets for community-based natural resource management in Bhutan (Gross National Happiness Commission 2008). Based on their abundance, easy propagation, multi-purpose use, and economic potential, *Dendrocalamus* spp. have been identified as one of the three bamboo taxa for NTFP development in the country (Social Forestry Division 2008). Shoots and culms are mainly used for domestic purposes at present, while commercial markets for most species and products are weakly developed.

Bamboo shoot production mainly depends on the harvesting regime (thinning of culms), as well as on water and nutrient supply (Kleinhenz and Midmore 2001). The only reasonably applicable bamboo management intervention in Bhutan is culm thinning, since irrigation, fertilizing, and mulching are highly labor and cost intensive, and their application would divert resources away from agricultural production. However, systematic clump management regimes are essential to prevent random harvesting, which results in a decline of clump productivity (Virtucio 2009). Harvesting regimes relevant in our context have been developed for *D. strictus* in India (Tewari 1992), as well as *D. asper* (Decipulo et al. 2009) and *Bambusa blumeana* (Marquez 2009; Malab et al. 2009) in the Philippines, with the aim of maintaining balanced age distributions of young (1- to 3-year-old) culms. Harvesting promotes clump regeneration: bamboo genets compensate for the loss of photosynthetically active tissue by mobilizing reserves from belowground rhizomes and allocating them to the production of new shoots. The majority of young shoots regenerate on rhizomes belonging to one- to two-year-old culms. Therefore, harvesting of these culms detrimentally affects regeneration (Malab et al. 2009), a trade-off which must be considered for bamboo shoot and culm production objectives.

In order to address the most important questions of community-based sustainable utilization of *D. hamiltonii* Munro var. *edulis* Munro, the most important bamboo species in southern Bhutan, the objectives of the present study were to (1) describe the state of local ecological knowledge (LEK) about *D. hamiltonii* Munro var. *edulis* Munro, and (2) test and evaluate harvesting methods designed by integrating LEK and science.

## Materials and Methods

### Species and Study Area

*D. hamiltonii* is a large sympodial bamboo with pachymorph rhizomes and culms, which grow up to 25 meters height (Stapleton 2000). The distribution of *D. hamiltonii* Munro (common name: Pakshing) ranges from the central Himalayas to northeast India (Troup 1921; Seethalakshmi and Kumar 1998), and includes the subtropical and warm-temperate broadleaf zones of Bhutan up to an altitude of 1800 m (Stapleton 2000). *D. hamiltonii* Munro var. *edulis* Munro is especially palatable with high nutritional value (Bhatt et al. 2005) and is common in central and eastern Bhutan (Stapleton et al. 1997). It occurs in open forests, frequently establishing after disturbances (Seethalakshmi and Kumar 1998) and is often cultivated. Thin-walled culms lead to flexibility, making it suitable as an all-purpose weaving material. Although thin walled, culms are also preferred as fencing material over other bamboo species because of their roughness and durability (Rai pers. comm.). The foliage is harvested to feed cattle and horses and is grazed by mithun (*Bos frontalis*) (Sundriyal and Sundriyal 2004). Shoots start to appear at the beginning of June, are harvested between mid-June and the end of August, and are consumed fresh, dried, shredded, or pickled.

The research site was located west of Tshanglajong village (27°06′27.54″N, 90°42′26.77″E) on an east-northeast facing slope at 870 m altitude with moderately moist site conditions. The village is located in Zhemgang district along the lower Mangduechu valley at an altitude ranging from 700 to 1000 m. Mean annual minimum and maximum temperatures measured at the Yebilaptsha climate station 2.6 km away are 15.2 and 26.2 °C, respectively. The mean annual precipitation is 1750 mm, the bulk of which falls during the summer monsoon period from May to September. The village was settled approx. 95 years ago, which is why we decided to use the term LEK instead of traditional ecological knowledge. The surrounding area is dominated by open *D. hamiltonii* forests (Rao and Saxena 1995). A community forest partially focusing on management of *D. hamiltonii* was incorporated in Tshanglajong a year after the start of the present study.

### Survey of Local Ecological Knowledge on Bamboo Ecology and Use

In an action research framework, social research methods were applied to gain insight into LEK on bamboo ecology, harvesting methods, utilization, socio-economic significance, and farmer’s objectives regarding bamboo use. Specific methods applied included semi-directive interviews, an analytical workshop and collaborative field work (Huntington 2000). All researchers in the field spoke the local language.

Semi-directive interviews were conducted to identify general patterns of LEK and local objectives of bamboo utilization. Invitations to attend a structured analytical workshop to triangulate interview findings and to define bamboo management objectives were sent to all households, and as habitual in Bhutan, one representative per household with the widest experience on the topic attended. Documentation of LEK focused on distribution, habitat characteristics, phenology, growth characteristics, age determination, morphology, yield, traditional harvesting techniques, utilization, income generation, and related employment, as well as legal and regulatory constraints related to bamboos and their utilization. Documented LEK was used to facilitate the identification of people’s goals regarding bamboo utilization and to design experimental treatments for bamboo harvesting focusing on these goals. Collaborative field work was conducted in the forest and helped verify the information obtained during the analytical workshop and gain practical insight into the subject. The approach also facilitated the development of ownership over the research and its results by users.

### Experimental Harvesting Methods and Data Collection

Experimental treatments were designed based on the requirements of local users in terms of labor input, its timing and the output of desired products. The experiment was established in spring 2009 with random selection of 16 bamboo clumps located in an area of approximately 0.25 ha to ensure homogenous environmental conditions. Selected bamboo clumps had no damage or signs of harvesting and had clearly defined clump edges. Treatments were defined by a combination of harvesting prescriptions for shoots and culms (Table 1) and applied randomly to selected clumps after initial measurements. The 16 bamboo clumps were evenly allocated to treatments and controls, such that four clumps received each of the three harvest treatments, and four clumps were reserved as controls.

**Table 1:** Harvest treatments applied to clumps of *Dendrocalamus hamiltonii*

Treatment	Shoots harvested (%)	Culms >2 years harvested (%)	Description
Control	0	0	No intervention
Selective cut	25	25	Removal of dead culms and stumps, harvest of shoots and culms from inside out
Horseshoe cut	75	75	Removal of dead culms and stumps, convex arch facing upslope, harvest of shoots and culms from inside of arch
Clear cut	50	100	Removal of dead culms and stumps, harvest of shoots from inside out

The three harvest treatments increased in intensity: selective cut, horseshoe cut, and clear cut. Selective cut was defined by low intensity removal of shoots and culms (25 % each). Since destruction of shoots near the perimeter of clumps may lead to clump congestion and ultimately to degradation (Troup 1921; Franklin 2008), selective harvest of culms started in clump centers. The horseshoe method is widely practiced in India and Nepal, and was applied orienting the convex arch of the shoe facing uphill in order to prevent accumulation of debris in the arch. With this method, new shoots are mainly added on the outer arch of the horseshoe, and therefore clumps are expected to expand uphill (Bradshaw 1997). Both shoots and culms were harvested at 75 % intensity with the horseshoe method. The clear cut treatment included the removal of 50 % of new shoots and all culms older than two years (Table 1). Culms were harvested above the second node, resulting in a minimal stump height of approx. 10–25 cm.

Monitoring and harvesting were carried out in seasons corresponding to roughly defined traditional harvesting seasons (summer for shoots, winter for culms). Monitoring consisted of enumeration of harvestable shoots and culms. Shoots and culms of no value to local users (dead, broken, undersized, not of the right age) were excluded from enumeration. Harvesting of culms was restricted to culms older than two years, as younger culms are of limited use, and because buds on their rhizomes form new shoots. Because the recruitment of *Dendrocalamus* varies greatly between years (Decipulo et al. 2009; Marquez 2009), harvesting and monitoring took place in 2009, 2010, and 2011. Additionally, culm dynamics were monitored until 2013, by enumerating shoot and culm recruitment. For each clump, we measured initial clump diameter, enumerated initial number of present-year and older live and dead culms, as well as the number of annually harvested shoots and culms.

### Data Analysis

Audio results of participatory exercises were not recorded, since this is not customary in Bhutan. Instead, the interviews were noted and compiled manually. Results were presented and verified in the analytical workshop, which was additionally used to reach a consensus on participants’ objectives regarding bamboo utilization.

For all experimental data, we initially computed descriptive statistics and the Pearson correlation coefficient for all pairs of variables. We then performed two preliminary linear regression analyses to illuminate the relationship between shoot recruitment in 2010 and (1) the total number of culms in 2009, and (2) the number of culms harvested in 2009. General linear mixed models (GLMMs) were then formulated to evaluate the effects of harvest treatments on the bamboo resource over the study period. We estimated models to quantify the effect of harvesting on (3) clump diameter, (4) productivity index (PI), defined as the ratio of shoot recruitment to the number of culms per clump (following Midmore 2009), (5) harvesting response, defined as the ratio of shoot recruitment to the number of harvested culms per clump, (6) shoot recruitment, (7) culm recruitment, (8) shoots harvested, and (9) culms harvested. GLMMs were formulated to include fixed effects for treatment, year as a repeated effect, and their interaction. In addition, to account for the potential influence of initial values, the analysis of clump diameter (3) included initial diameter (in 2009) as a covariate. The productivity analysis (4) included the current-year culm production as a covariate. The analyses for shoot and culm recruitment (6 and 7) and harvest (8 and 9) included the cumulative harvest as a covariate. Because data from the same clump are likely more correlated than those from different clumps, these GLMMs included a random effect for clumps with a compound symmetric error structure to account for the correlation among shoots in the same clump. A Kenward–Roger approximation was used to adjust the degrees of freedom as suggested for mixed models (Kenward and Roger 1997). To meet the assumptions of normality and homoscedasticity, a square root transformation was performed for harvesting response, culm recruitment, and culms harvested. A log-transformation was performed for PI. All analyses involving harvesting data (harvesting response, shoots harvested, and culms harvested) were performed on treated data only (i.e., all control data were excluded, since no harvest occurred).

Shoot and culm harvest were identified to be equal management priorities (see Results). Thus, we also analyzed harvest data pooled over three years in order to evaluate differences in shoot and culm harvest over the entire period simultaneously (analysis 10). This resulted in dependent variables for combined shoot harvest (2010 and 2011), and combined culm harvest (2009, 2010, and 2011), which could be analyzed in a fixed-effects ANOVA framework. Since the simultaneous assessment of numbers of shoots and of culms represents a multivariate question involving the interaction and trade-off between two response variables, we performed a multivariate ANOVA (MANOVA; 10) rather than separate univariate ANOVAs (Scheiner 2001). As the control treatment did not include harvest by definition, data from these clumps were excluded from the analysis. As in the univariate analyses, the initial clump diameter and the initial number of culms in 2009 were included as covariates in analyses as proxies for initial clump size, resulting in a multivariate analysis of covariance (MANCOVA).

Since appropriate interpretation of GLMMs, MANCOVA, and ANOVA results requires normality and homoscedasticity of residuals, these were verified by visual examination of residual graphs. Where significant differences by treatment were indicated (*p* ≤ 0.05), a Scheffé test was performed on univariate estimated marginal means. Where appropriate, marginal means were back-transformed, taking into account bias introduced by log-transformation (sensu Sprugel 1983). Where a significant treatment by year interaction was detected, the simple effect of treatment within each year was tested via tests of simple effects (Winer 1971). Multivariate differences in response variables were evaluated via Wilks’ *λ*, which is a multivariate F test of the ratio of the variance/covariance matrices of the errors versus the effects. However, because these means are not adjusted for the correlation between dependent variables in the MANCOVA, we then performed multivariate comparisons among treatments via orthogonal contrasts. All analyses were conducted using SAS (version 9.2) via procedures PROC REG, PROC MIXED, and PROC GLM (SAS Institute Inc. 2002–2008).

## Results

### Local Ecological Knowledge and Local Users’ Objectives

Of the six species of bamboos growing in and around Tshanglajong, local users reported that *D. hamiltonii* was the only species used for a wide variety of purposes, such as fencing, weaving, and construction. Local users observed that *D. hamiltonii* grows best in valleys and depressions and in general under open forest canopy. They had encountered sporadic, but no mass flowering of *D. hamiltonii*, followed by monocarpic die-off of clumps. Local users did not apply specific methods of harvesting, nor did they apply local restrictions on collection time; rather, they harvested shoots and culms whenever available or required. Stump height was determined by ease of cut and was usually higher towards clump centers and in highly congested clumps. Easily accessible culms of suitable quality, usually near to clump edges, were reportedly harvested on a preferential basis. Collection time for shoots, of which the top 30-40 cm are harvested, was determined by their emergence, generally in July and August. Harvesting of culms was mostly done during the dry winter season to prevent borer damage.

The assignment of culms to different uses depended on physical qualities, which are a function of culm age. Users were confident to determine culm age up to three years based on morphological properties. Young culms were used for weaving, while older ones were used for construction purposes. Products were either sold locally, in the nearest town at eight km distance, or the district headquarters at 42 km distance. Bamboo culms were sold infrequently. Parts of the population reported occasionally engaging in weaving of mats and baskets for domestic as well as commercial purposes. In spite of its abundance, however, only two local users were intensively involved in bamboo harvesting and processing. People were interested in harvesting both shoots and culms, but did not want to devote much time and resources towards bamboo activities due to unreliable markets.

### Bamboo Clump Properties

**Fig. 1:**
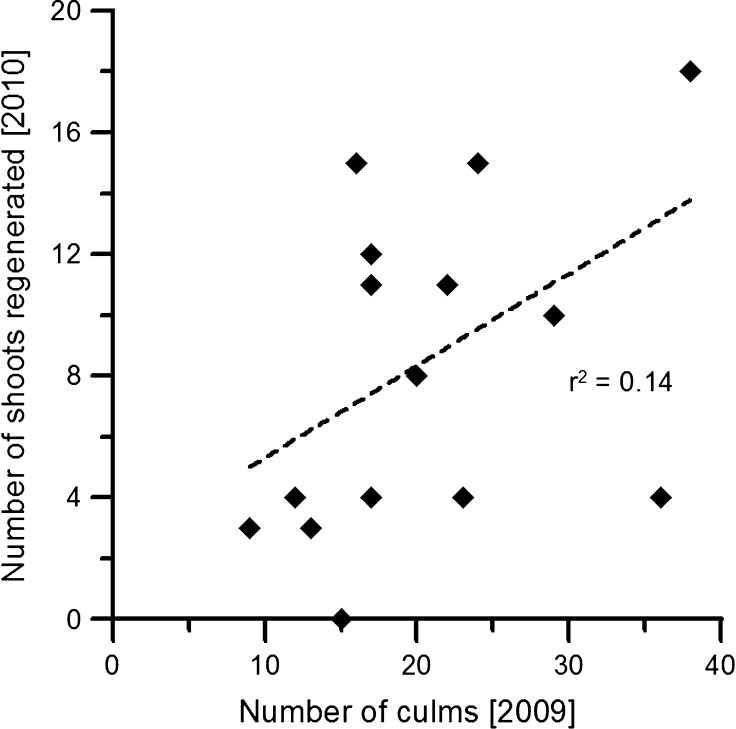
Relationship between number of culms per clump and number of shoots emerged the following summer

**Fig. 2:**
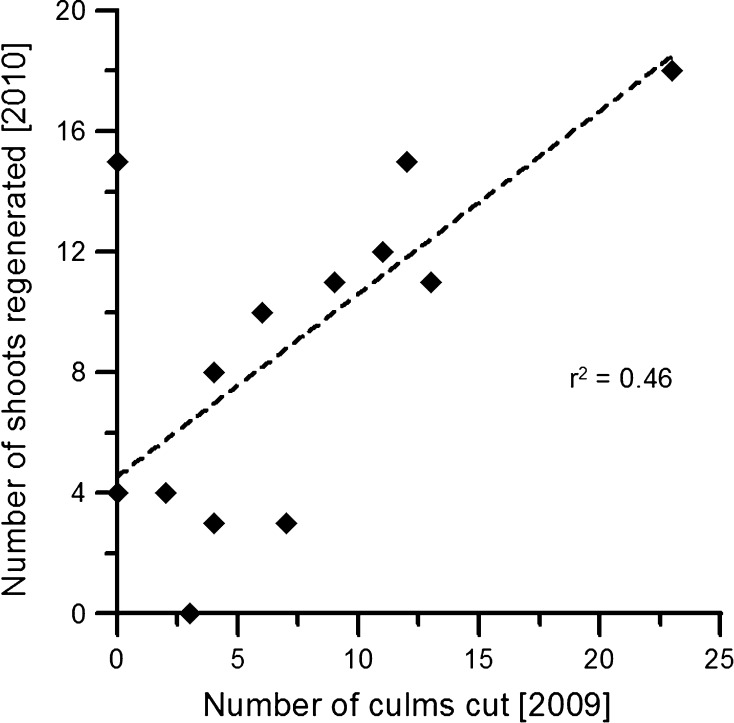
Relationship between number of culms harvested per clump and number of shoots emerged the following summer

The relationship between the number of culms in 2009 and the number of shoots regenerated per clump in 2010 was weak (*r*^2^ = 0.14, Fig. 1). On the other hand, the relationship between the number of culms harvested per clump in 2009 and the number of shoots regenerated in 2010 was stronger and significant (*r*^2^ = 0.46, Fig. 2).

Prior to application of treatments in 2009, mean clump diameter was 308 cm, and on average a clump had 21 culms with a median culm diameter of 8.56 cm (Table 2). About 15 % of culms were dead, while 13 % were current-year culms. Over the course of the experiment, neither time nor harvesting treatments nor their interaction showed significant effects on clump diameter (Table 3, Analysis 3, *p* > 0.05).

**Table 2:** Bamboo clump characteristics prior to application of treatments in 2009

Parameter	Mean	Standard error
Clump diameter (cm)	308.44	74.68
Total number of culms	20.75	8.16
Median culm diameter (cm)	8.56	0.89
Proportion of current-year culms	0.126	0.107
Proportion of dead culms	0.151	0.105

**Table 3:** GLMM Type III test of fixed effects of various *Dendrocalamus hamiltonii* response variables to experimental harvest treatments, year, and initial conditions

Response variable	Source	Num DF	Den DF	*f* value	Pr > *F*
3) Clump diameter	Treatment	3	11	2.99	0.0775
	Year	2	24	0.59	0.5615
	Treatment*year	6	24	1.90	0.1228
	Initial clump diameter	1	11	12.11	**0.0051**
4) Productivity index	Treatment	3	12.3	3.81	0.0389
	Year	2	23.8	26.05	<0.0001
	Treatment*year	6	23.7	2.55	**0.0473**
5) Harvesting response	Treatment	2	8.39	3.03	0.1024
	Year	2	17.8	10.19	0.0011
	Treatment*year	4	15.5	3.68	**0.0271**
	Current culm production	1	16.9	12.13	**0.0029**
6) Shoot recruitment	Treatment	3	11.7	4.71	0.0222
	Year	2	28.6	25.46	<0.0001
	Treatment*year	6	24.6	2.89	**0.0283**
	Cumulated harvest	1	14.6	25.41	**0.0002**
7) Culm recruitment	Treatment	3	10.1	8.95	0.0034
	Year	3	36.5	36.16	<0.0001
	Treatment*year	9	32.7	3.77	**0.0024**
	Cumulated harvest	1	45.3	3.85	0.0559
8) Shoot harvest	Treatment	2	8.02	2.36	0.1561
	Year	1	9.33	14.64	**0.0038**
	Treatment*year	2	9.11	4.01	0.0563
	Cumulated harvest	1	8.08	61.41	**<0.0001**
9) Culm harvest	Treatment	2	2.66	2.56	0.2405
	Year	2	11.7	16.59	**0.0004**
	Treatment*year	4	7.98	2.93	0.0914
	Cumulated harvest	1	25	0.09	0.7623

Bold values indicate significant interactive effects, or in their absence significant individual effects

### Productivity Index and Harvesting Response Under Experimental Treatments

**Fig. 3:**
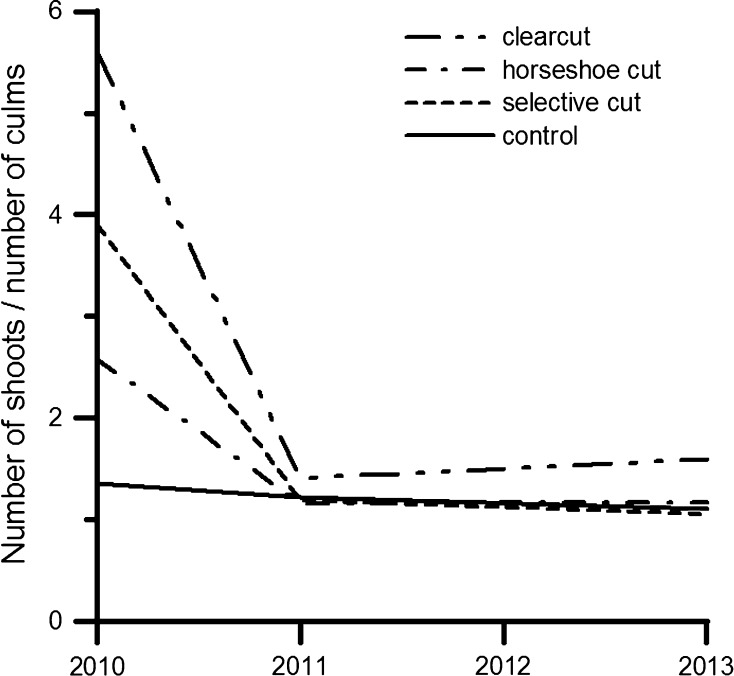
Productivity index under three harvest and control treatments

We observed significantly different Productivity Indices among treatments by year (Table 3, Analysis 4, *p* ≤ 0.05). Tests of simple effects showed that in 2010 the control treatment had a significantly lower proportion of shoots, as compared to harvest treatments. Clear cut and horseshoe cut treatments also differed significantly from each other in the same year. PI showed a significant decline over time irrespective of the type of harvest, but this decline was not observed in the control treatment. From 2011 onwards, there was no significant difference among treatments (*p* ≤ 0.05; Fig. 3).

**Fig. 4:**
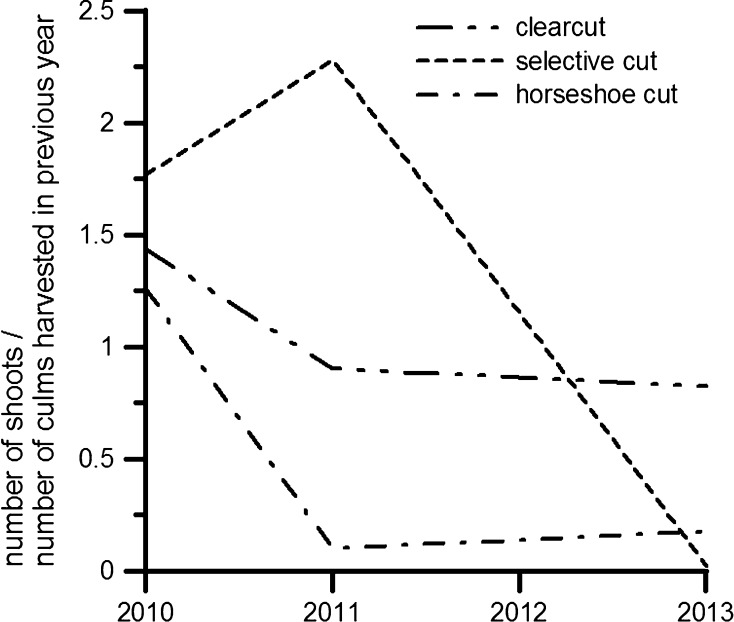
Harvesting response over time under three harvest treatments

The harvesting response also differed significantly among treatments by year (Table 3, Analysis 5, *p* ≤ 0.05). Harvesting response was significantly higher with selective cut as compared to horseshoe cut in 2011 and with clearcut as compared to selective cut in 2013 (*p* ≤ 0.05; Fig. 4). In the first year after harvest, all treatments led to greater recruitment of new shoots as compared to the number of culms harvested in the previous year. By the end of the observation period, this ratio fell below the level of potential self-replacement with all treatments (Fig. 4).

### Recruitment and Harvest of Shoots and Culms Under Experimental Treatments

**Fig. 5:**
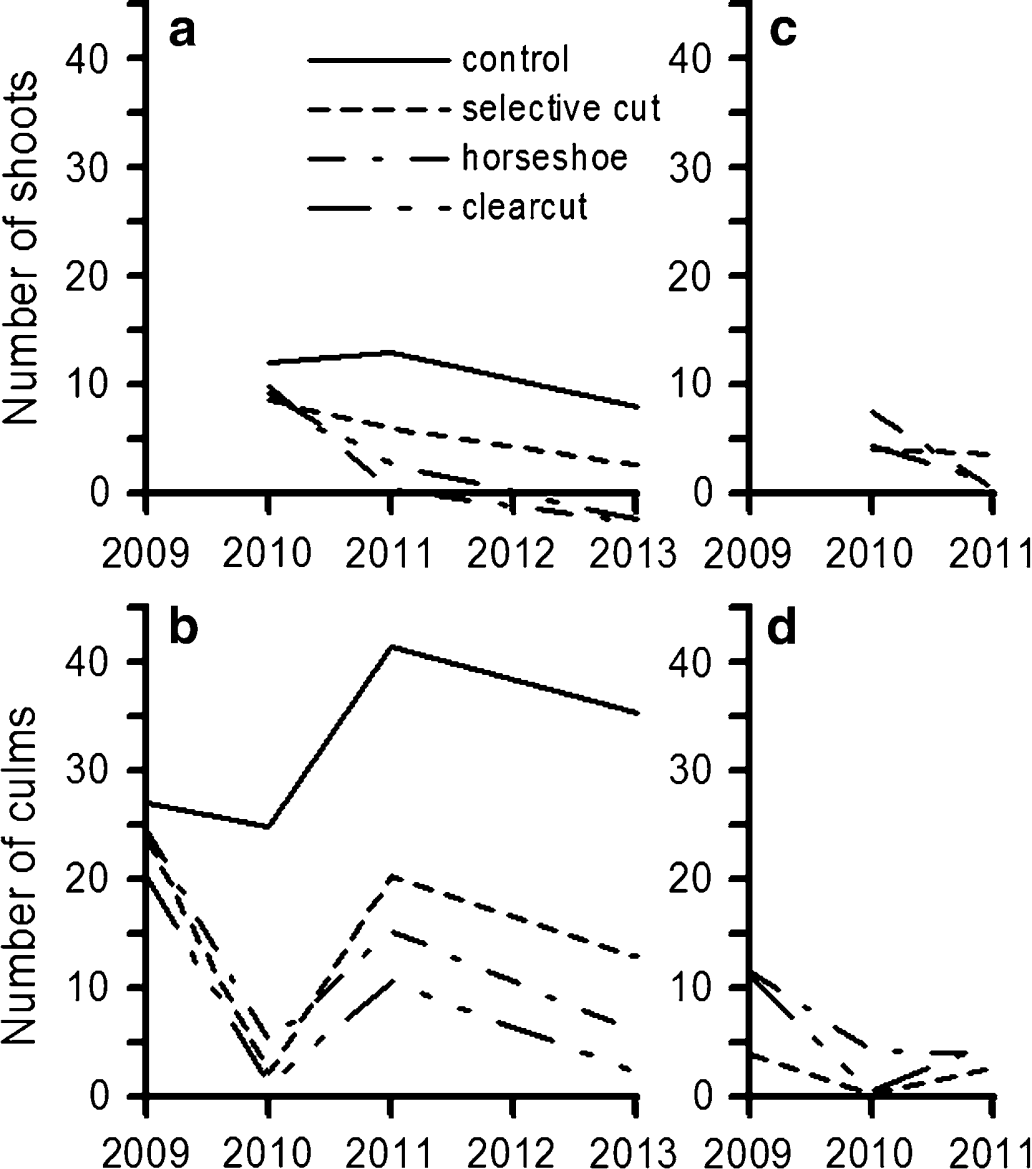
Number of shoots recruited (*a*), culms recruited (*b*), shoots harvested (*c*), and culms harvested (*d*) under different treatment regimes over time

While recruitment and harvest of shoots constantly declined over time irrespective of treatments, these negative trends were less consistent and showed fluctuations in the case of culms. Recruitment generally decreased with the intensity of treatments and harvested output did not differ among treatments (Fig. 5).

There was a significant time x treatment interaction for shoot recruitment (Table 3, Analysis 6, *p* ≤ 0.05). A test of simple effects confirmed differences in shoot recruitment between control and the rest of the treatments in 2011 and 2013 (*p* ≤ 0.05). Declining shoot recruitment was confirmed by significant differences among years for all treatments but control (Fig. 5a). Similarly, there was a significant time x treatment interaction on culm recruitment (Table 3, Analysis 7, *p* ≤ 0.01), and tests of simple effects confirmed increasingly diverging mean culm numbers between treatments. The culm population of the control treatment was significantly higher than of other treatments from 2010 onwards. Additionally, the culm population of selective cut was significantly higher than of clearcut in 2013 (*p* ≤ 0.05; Fig. 5b). Time but not harvest treatment had a significant effect on the number of shoots harvested (Table 3, Analysis 8, *p* ≤ 0.01), which showed a significant decline from 2010 to 2011 (*p* ≤ 0.05; Fig. 5c). Similarly, only time showed a significant effect on the number of harvested culms (Table 3, Analysis 9, *p* ≤ 0.001). From 2009 to 2010 culm harvest showed a significant decline and from 2010 to 2011 and significant increase (*p* ≤ 0.05; Fig. 5d).

**Fig. 6:**
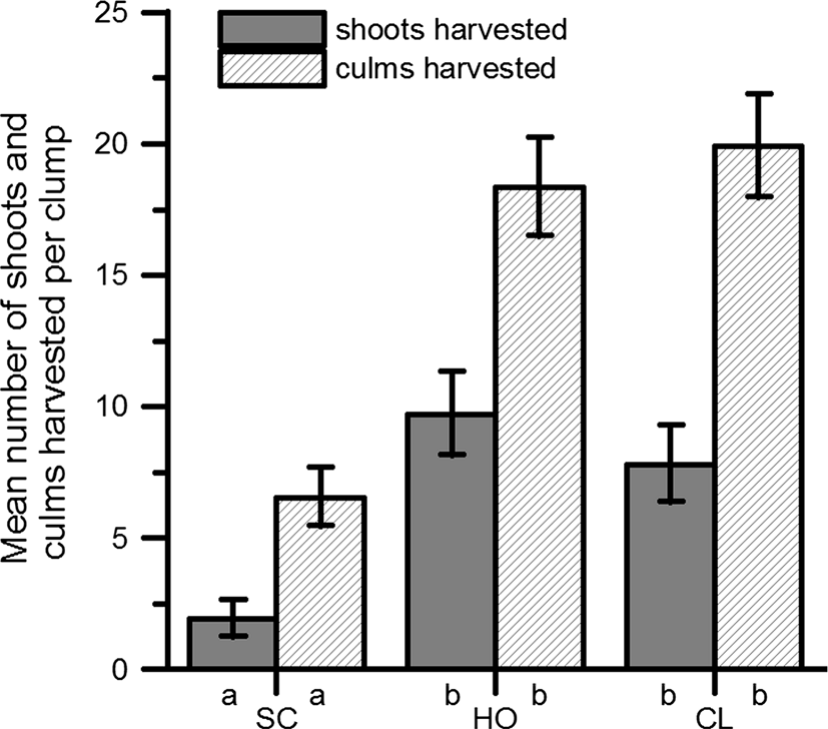
Total number of shoots and culms harvested per *Dendrocalamus hamiltonii* clump under different harvest regimes (estimated marginal mean ± SE resulting from MANCOVA; *SC* selective cut, *HO* horseshoe cut, *CL* clear cut)

The multivariate test of differences among harvest treatments was statistically significant (Analysis 10, Wilk’s *λ* = 0.128, *p* ≤ 0.01), and the model explained a large proportion of the variation of the response variables. The number of culms before application of treatments was a significant covariate in the multivariate model (Wilk’s *λ* = 0.173, *p* ≤ 0.01) and was significant in both separate univariate GLMMs (Table 4). The number of shoots and culms harvested was significantly lower with selective cut as compared to the other two treatments (Fig. 6; contrasts, *p* ≤ 0.05).

**Table 4:** MANCOVA Type III test of fixed effects for *Dendrocalamus hamiltonii* shoots harvested per clump, and culms harvested per clump as dependent variables

Response variable	Source	DF	Type III SS	Mean square	*F* value	Pr > *F*
Shoots harvested per clump	Treatment	2	171.40	85.70	9.59	**0.0075**
	Number of culms 2009	1	186.78	186.78	20.91	**0.0018**
Culms harvested per clump	Treatment	2	304.63	152.31	23.41	**0.0005**
	Number of culms 2009	1	247.44	247.44	38.03	**0.0003**

Bold values indicate significant interactive effects, or in their absence significant individual effects

### Practical Experiences with Experimental Harvesting

Users considered clear cut most easy to apply, but the lack of sustainability of this method was imminent to them. The initial implementation of the horseshoe harvest required the greatest effort in terms of labor input. Difficult situations arose when interlocking bamboo culms under tension had to be cut and carefully extracted from among the culms, which were retained. Subsequently, the horseshoe method proved easier to administer, as culms and shoots were readily accessible for harvesting from clump edges. Selective cut was initially easier to implement as compared to the horseshoe cut, but subsequently its implementation was more difficult. Cutting of culms with low stumps in clump centers was difficult and frequently dangerous, despite reduced culm densities. Situations where harvested culms had to be extracted from among interlocking culms under tension were common and frequently led to damaged culms. Overall, users preferred the application of the horseshoe method.

## Discussion

### Experimental Design Based on Users’ Objectives

LEK is believed to promote sustainable utilization of natural resources, particularly where scientific knowledge is lacking (Berkes et al. 2000). In our case, local users had rich LEK on growth conditions and other aspects necessary to implement the extensive, very low intensity harvesting, which was the traditional resource management system for *D. hamiltonii*. At the same time, they lacked knowledge on more intensive harvest of the species, which aims at producing more diverse products, reflecting changing local socio-economic priorities. This study tapped into LEK of forest users to design more intensive harvest methods. Local users were interested in extensive clump management practices leading to the simultaneous maximization of harvestable number of shoots and culms, which has also been confirmed by other studies in the same locality (Trinh Thang et al. 2011). Due to financial and labor implications, we thus had to rule out irrigation and fertilization as possible treatments in the trial. The lack of markets has also been identified independently as the reason for low interest of local users to devote more resources towards bamboo management (Trinh Thang et al. 2011).

In this study, we included three different harvest treatments and a non-harvest control for comparison. The simultaneous interest in shoot and clump production helped define two intermediate intensity treatments, and clear cut was included as the most intensive harvest treatment. Cleaning of dead culms and stumps was included in all harvest treatments to reduce clump congestion and to ease harvest as stated by local users. The selective cut treatment was designed as a low-input thinning without spatial regulation of culms to be removed. The horseshoe cut method (Tewari 1992; Bradshaw 1997) was included as a more labor-intensive alternative, requiring skills in proper spatial arrangement of culms to be removed. We expected that shoots located closer to the edge of clumps were less likely to survive (Franklin 2008), in which case the horseshoe method would have provided low recruitment and yield of shoots and culms. Bamboo shoots have been reported to be heavily browsed by wild animals and destroyed by insects (Taylor and Zisheng 1987), and this was also observed with *D. hamiltonii* in our study site (Trinh Thang and Dorji 2010). The clear cut method was an alternative, which can be most easily and safely implemented in unmanaged, congested clumps of *D. hamiltonii*; where clumps are unmanaged, most culms are interlocking and under tension, which leads to dangerous situations during harvesting. Maintenance of a balanced age distribution with removal of older culms is generally deemed beneficial for clump vigor (Malab et al. 2009; Nath and Das 2011), but our study design did not allow for testing the effects of different age distributions on productivity.

According to the objectives defined by local users, we applied harvest treatments annually. The rationale for winter harvest of culms is scientifically proven, as the starch content of culms at this time is very low, making them unattractive to insects (Dransfied and Widjaja 1995). Local users preferred the horseshoe harvest method based on the yields of desired products and practical considerations of ease and safety of harvesting. Nevertheless, our results clearly indicated that annual harvest according to any of the applied methods was not sustainable. With the more intense methods of horseshoe cut and clear cut, shoot recruitment declined to negligible levels. Selective cut on the other hand maintained relatively stable culm populations and 4 years after the start of harvest still showed considerable recruitment of new shoots. Considering the combined output in terms of shoots and culms, this last method, however, produced significantly lower amounts as compared to the previous two. Investigation on the management of *D. strictus*, which is a close relative of *D. hamiltonii*, revealed that sustainable harvest depends on felling intensity, cutting methods, and felling cycle. Recommendations include a 3- to 4-year felling cycle with retention of new culms along with a certain number of old culms (Tewari 1992). In our case, continued annual removal of shoots and culms did not leave sufficient time for bamboo clumps to regenerate, necessitating further studies to investigate appropriate cutting cycles.

### Characteristics of Clump Regeneration

The PI indicates the rate of vegetative regeneration of the bamboo clump. Other studies on harvesting large, clump-forming bamboos report a close correlation between the number of culms (Vázquez-López et al. 2004)—specifically the number of current-year culms (Malab et al. 2009)—and shoot production (PI). Even though we found indication for the above relationships to possibly hold true for *D. hamiltonii*, the number of culms harvested more closely influenced the number of shoots recruited in the following season (harvesting response). Although successful recruitment of shoots to culms may be limited (Franklin 2008), this relationship can be explained by the compensatory growth mechanism (McNaughton 1983), according to which bamboos compensate harvesting losses with increased growth of new shoots. Reduced inter-culm competition also promotes the regeneration of new shoots (Nath et al. 2006), possibly explaining why our PI initially increased with increasing intensity of the harvesting treatment. While belowground reserves stored in bamboo rhizomes are substantial, they are nevertheless a minor contributor to aboveground growth, which is mainly driven by photosynthesis of one-year leaves (Li et al. 1998). As a result, our treatments defined by a greater intensity of shoot harvest led to a greater decline in the recruitment of new shoots. At a nearby study on intensive management of *D. hamiltonii* clumps for bamboo shoot production, Trinh Thang and Dorji (2010) found comparable numbers of culms per clump and proportions of shoots (PI = 0.17) in untreated clumps, as reported in the present study for untreated clumps (0.2).

### Shoot and Culm Recruitment and Harvesting Under Different Treatments

Annual application of harvest treatments exceeded the level of self-replacement, as indicated by the decline in shoot recruitment, and this needs to be considered when designing cutting cycles. Apparently, the harvest simulated with our experimental treatments was not sustainable. In spite of the continuous application of different levels of shoot removal, we did not find significant differences in total shoot recruitment between treatments, due to compensatory growth (Vázquez-López et al. 2004; Decipulo et al. 2009). We did not find evidence for clump congestion restricting shoot recruitment (Decipulo et al. 2009; Malab et al. 2009), as shoot recruitment was highest in the control treatment throughout the study.

The high combined harvested output in shoots and culms with the horseshoe cut was likely a result of increased edge length of clumps and decreased distance of culms from the clump edges. Horseshoe cut avoids congestion of clumps, and leads to improved clump vigor, manifested through increased shoot recruitment (Malab et al. 2009; Franklin 2008). The high combined harvest output in the clear cut treatment is likely a result of compensatory growth response to virtually complete removal of photosynthesizing biomass (McNaughton 1983). While a trade-off between shoot and culm production was observed with *D. asper* (Decipulo et al. 2009), this was not observed in our case.

## Conclusions

Before markets are readily available for bamboo products, local users have low willingness to invest into management of *Dendrocalamus hamiltonii*. The notable exceptions are silvicultural interventions, resulting in the harvest of shoots and culms, both for domestic consumption and for limited sale. Local ecological knowledge alone may not provide answers to the sustainable utilization of NTFPs under previously unprecedented, more intensive management. As a result, we developed and tested silvicultural methods in a participatory action research framework integrating local ecological knowledge. Tested silvicultural methods lead to high yield of shoots and culms in the first years of harvesting. Weaker intensity selective cut appeared to be sustainable until the third year of consecutive harvesting, while higher intensity horseshoe cut and clear cut yielded more shoots and culms. However, annual harvest over several years jeopardizes clump productivity. The horseshoe cut method was easy to administer after initially greater investment of labor and maximized safety of work while harvesting. The study highlighted the need to follow harvesting experiments for a number of years to avoid drawing early conclusions and the need to conduct research on longer cutting cycles.
